# Prognostic impact of CD168 expression in gastric cancer

**DOI:** 10.1186/1471-2407-11-106

**Published:** 2011-03-24

**Authors:** Sumiya Ishigami, Shinichi Ueno, Yuka Nishizono, Masataka Matsumoto, Hiroshi Kurahara, Takaaki Arigami, Yasuto Uchikado, Tetsuro Setoyama, Hideo Arima, Kita Yoshiaki, Yuko Kijima, Masaki Kitazono, Shoji Natsugoe

**Affiliations:** 1 Digestive Surgery Surgical Oncology, Kagoshima University School of Medicine, 8-35-1 Sakuragaoka Kagoshima 890-8520, Japan

## Abstract

**Background:**

Interactions of stromal hyaluronic acid (HA) with its binding protein RHAMM (receptor for HA-mediated motility) (CD168) have been reported to affect tumor extension and the migration of crucial molecules to promote tumor progression and metastases. Cancerous CD168 expression is correlated with aggressive biological features in several cancers. However, the clinical implications of CD168 positivity in gastric cancer have remained unclear.

**Methods:**

We examined the CD168 expression of 196 consecutive gastric cancer patients by immunohistochemistry. According to CD168 positivity, the 196 gastric cancer patients were divided into two groups (57 CD168-positive and 139 CD168-negative patients). The correlation between CD168 expression and clinicopathological factors (age, sex, histology, tumor depth, lymph node status, and vessel invasion) was evaluated according to the Japanese Classification of Gastric Carcinoma.

**Results:**

Cancerous CD168 expression was detectable in 57 of the 196 tumors (29%). CD168 positivity was significantly correlated with the depth of invasion, nodal involvement, and vessel invasion (p < 0.01). Survival analysis of the 196 gastric cancer patients showed that the CD168-positive group had a significantly higher mortality than the CD168-negative group (p < 0.01). In terms of a correlation with CD168 positivity at separate clinical stages, a significance difference was only found in stages II and III. Multivariate analysis revealed that CD168 expression was a significant independent prognostic marker (p = 0.013) after depth of invasion (p < 0.005) and nodal involvement (p < 0.01).

**Conclusion:**

Our results suggest that cancerous CD168 positivity is strongly related to the invasion and metastasis of gastric cancer tumors. These results suggest that cancerous CD168 expression can be used as a prognostic marker of gastric cancer owing to its interactions with stromal hyaluronic acid.

## Background

Hyaluronic acid (HA) is a component of the extracellular matrix. In cancerous tissue, HA is abundantly secreted from stromal fibroblasts in response to humoral factors derived from tumor cells [1]. It is associated with breast cancer progression. Recently, HA receptors on tumor cells have been detected. CD44 and intracellular hyaluronic acid binding protein (RHAMM/IHABP) (CD168) [2,3] are representative HA receptors, which have been identified as members of the microtubule-associated protein (MAP) family. Another of the HA receptors, CD168, was isolated from a culture supernatant of RAS-transformed murine 3t3 fibroblasts [4]. CD168 on tumor cells is stimulated by HA and activates intracellular kinase cascades. This stimulates microfilament formation in tumor cells and promotes cellular motility. In vitro, RHAMM is upregulated in many tumor cell lines, and its expression is essential for their continued tumorigenicity and metastasis [5,6]. Clinically, CD168 expression is found in malignant glioma [7], breast [8,9], urinary bladder [10], and endometrial cancers [11].

Lugli et al. immunohistochemically investigated the CD168 positivity of tumor cells and identified a significant correlation with biological aggressiveness in colorectal cancer [12]. However, the significance of CD168 in the prognosis of gastric cancer is not fully understood. In the current study, we attempted to clarify the clinical features of CD168-positive gastric cancer, and the clinical implications of CD168 expression were discussed.

## Methods

One hundred and ninety-six consecutive gastric cancer patients who underwent R0 resection at Kagoshima University Hospital between 1998 and 2004 were enrolled in the present study. The patient group was composed of 135 males and 61 females, ranging in age from 43 to 87 years (mean 63 years). No patients received preoperative chemotherapy. A total of 107 underwent a distal gastrectomy, 66 underwent a complete gastrectomy, and the remaining 23 underwent a proximal gastrectomy. All patients underwent R0 resection with more than D1 lymph node dissection. After the final pathological evaluation, 89, 27, 43, and 37 patients were classified with stage I, II, III, and IV gastric cancer, respectively (Additional file 1). Clinical factors were assessed using the Japanese Classification of Gastric Carcinoma [12]. This study was approved by the Ethical Committee of the University of Kagoshima, and written informed consent was obtained from all individuals.

### CD168 expression in gastric cancer by immunohistochemistry

**Figure 1:**
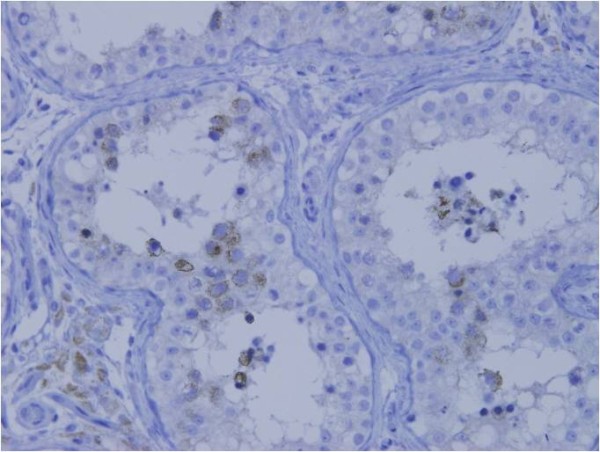
**Membranes of spermatocytes in seminiferous cells were positive for CD168**.

**Figure 2:**
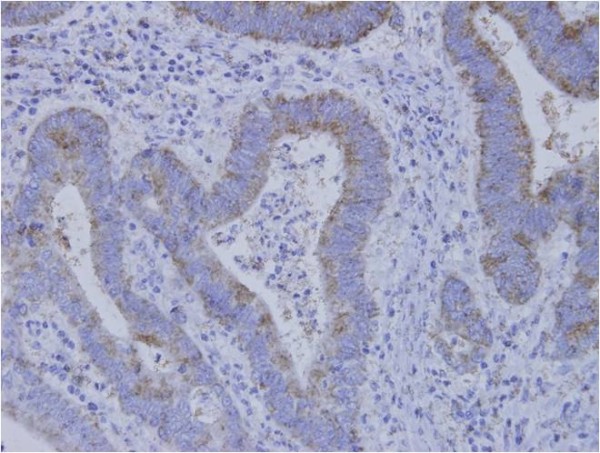
**CD168 positivity was identified not only at the cellular membrane but also in the cytoplasm of gastric cancer cells**.

Cancerous CD168 expression was assessed in accordance with previous reports [13] and visualized by avidin biotin complex (ABC) immunohistochemistry. Specifically, paraffin-embedded sections (4 μm), including tumor nests, were obtained from the 196 gastric cancer patients, and deparaffinized and soaked in phosphate-buffered saline (PBS) prior to immunohistochemical analysis. Sections were treated with 3% H2O2 for 30 minutes in order to block endogenous tissue peroxidase, followed by treatment with bovine serum for 30 minutes in order to reduce nonspecific binding. CD168 monoclonal antibody (2D6, Abcom, Japan) was diluted at 1:200 with PBS and incubated with the sections overnight at room temperature. Sections were rinsed in PBS and visualized using standard techniques for labeled avidin-biotin immuno-peroxidase staining. The membranes of spermatocytes in seminiferous cells were used as a positive control of the immunohistochemical staining, and found to be positive for CD168 (Figure 1). The CD168 positivity was observed not only at the cell membrane but also in the cytoplasm of gastric cancer cells (Figure 2). CD168 was also occasionally identified in non-cancerous gastric mucosa.

### Evaluation of CD168 positivity in gastric cancer

CD168 positivity in gastric cancer was evaluated in accordance with a previous report [13]. Specifically, when CD168 positivity was identified in cancerous tissue, the tissue was evaluated in 25 representative high-power fields (× 400) not only at each tumor nest but also at the invasive front of the tumor. All immunostained slides were evaluated by two independent observers (SI and AT), who were unaware of the clinical data or disease outcome. In accordance with a previous report [12], if cancerous CD168 positivity was identified, the patient was regarded as CD168 positive. Patients were divided into two groups according to CD168 positivity. The correlation between clinicopathological factors and CD168 expression was investigated.

### Statistical analysis

Statistical analysis of clinical features was performed by the χ2-test. Cumulative survival curves were drawn by the Kaplan-Meier method and statistical significance was calculated using the generalized Wilcoxon method. The Cox proportional hazard model was used in the multivariate analysis to determine independent prognostic factors. A p value of less than 0.05 was considered as statistically significant. The statistical analysis was performed using StatView ver.5 for Windows software.

## Results

### 1) Identification of CD168-positive cancer cells in gastric cancer and evaluation of CD168 positivity

CD168 positivity was identified not only at the cellular membrane but also in the cytoplasm of gastric cancer cells. Normal gastric glands showed partial positivity of CD168 (Figure 2). The 196 patients were divided into two groups, namely, 57 were placed in the CD168-positive group and 139 were in the CD168-negative group.

### 2) Clinicopathological features and CD168 positivity in gastric cancer

**Figure 3:**
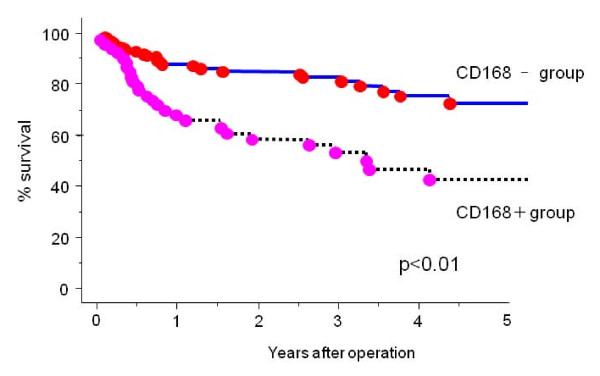
**The five-year survival rate of the CD168-positive group was 41%, which was significantly worse than that of the CD168-negative group (p < 0.01)**.

The correlation between CD168 expression and clinicopathological features that affected patient outcome was analyzed. CD168 positivity significantly correlated with deeper tumor invasion (p < 0.01), presence of lymph node metastasis (p < 0.01), lymphatic invasion (p < 0.05), and venous invasion (p < 0.05). There was no correlation between tumor histology and CD168 expression (Additional file 2). The five-year survival rate of the CD168-positive group was 41%, which was significantly worse than that of the CD168-negative group (p < 0.01) (Figure 3).

**Figure 4:**
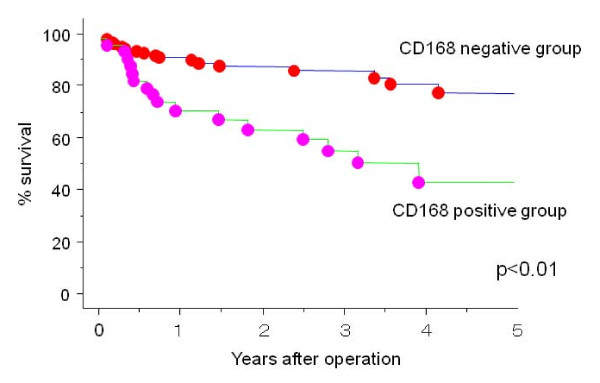
**Significant prognostic difference of CD168 positivity in stage II and III gastric cancer patients (p < 0.01)**.

In terms of respective clinical stages, there were only significant differences in CD168 positivity in stage II and III gastric cancer patients (p < 0.01) (Figure 4). Five clinical factors, including age, gender, depth of invasion, nodal involvement, and CD168 positivity, that affected patient outcome by univariate analysis were evaluated by multivariate analysis; cancerous CD168 expression was revealed as a significant prognostic factor after depth and nodal involvement (Additional file 3).

## Discussion and Conclusions

Several studies have identified a correlation between cancerous CD168 expression and tumor progression or high metastatic potential in breast [8], pancreas [14], and endometrial [11] cancers not including gastric cancer. As for the CD168 expression of gastric cancer, Li et al. [15] undertook the first detailed investigation and determined that intracellular CD168 levels increased with tumor progression, which is similar to our result. However, they could not clarify the clinical impact of CD168 expression or the prognostic value for gastric cancer because the number of gastric cancer patients was too small. Therefore, this is the first report to determine the correlation between CD168 expression and clinical factors in gastric cancer.

CD168 positivity was identified in cellular mucosa and cytoplasm in gastric cancer cells, similar to that in other types of tumors. Reportedly, RHAMM has at least two distinct functions and is distributed in both cytoplasm and nucleus. It functions as a cell-surface receptor for HA and a centrosomal protein that maintains the stability of the mitotic spindle [16]. However, we could not identify nuclear CD168 positivity in cancer and normal gastric tissues by immunohistochemical methods.

The proportion of CD168-positive gastric cancer cells was 28%. CD168 positivity was found to be 65% in esophageal squamous cell cancer [5] and 40% in colorectal cancer cells [13]. Thus, compared with other cancers, gastric cancer positivity appears to be relatively low, and may depend on cancer specificity. In normal gastric mucosa, CD168 was rarely expressed in the crypts, in contrast to that in colorectal mucosa. The frequency of CD168 positivity in normal gastric mucosa did not reach 10% according to Li et al. In this context, the independent effect of the CD168 positivity of gastric mucosa appears to be lower than that in colorectal normal tissue, which differed from the weak and diffuse CD168 expression in normal colon mucosa [13]. In the current study, we showed that the presence or absence of CD168 expression in gastric cancer directly affected clinical factors. In contrast, in colorectal cancer, CD168 expression was not found to be strongly independently associated with tumor progression and risk of tumor recurrence, although researchers insisted on the clinical merit of quantitative evaluation of CD168 positivity.

CD168 expression in gastric cancer was found to be significantly inversely correlated to not only tumor extension and nodal involvement but also surgical outcome in the present study. This agreed with findings for other types of tumor such as breast, colon [17], and prostate cancer. This suggests that CD168 overexpression may independently predict high metastatic potential in several carcinomas.

Zlobec demonstrated that cancerous CD168 expression was a strong prognostic marker in combination with p21 expression [18] or CD8-positive lymphocytic infiltration [19]. In this context, cancerous CD168 positivity independently affecting patient outcome via HA-related aggressiveness can act as a prognostic marker stratifying other prognostic markers in gastrointestinal carcinomas.

In the current study, we did not detect a significant correlation between tumor histology and cancerous CD168 expression. In breast cancer, Assmann reported that tumors of the lobular type exhibited a stronger expression of CD168 than carcinomas of the ductal type [20]. Moreover, CD168 expression in endometrial cancer was found to be correlated with histological grade of tumors [11], so CD168 expression may reflect histological aggressiveness. It is well known that gastric cancer has a diverse histology and exhibits a mixture of different types of histology in the same cancer nest. This may explain why CD168 expression was not correlated with tumor histology in gastric cancer.

It has been reported that p53 represses RHAMM expression and HA-dependent signaling and metabolism are controlled by p53 [21]. Moreover, Lin et al. showed that the androgen receptor regulates CD168 expression, and that CD168 is involved in downstream signaling in prostate cancer and acts as a native androgen to significantly promote the tumorigenicity of androgen-dependent prostate cancer cell lines [22]. Cancerous CD168 expression may reflect integration of biological behavior from hormonal and cell-cycle molecular conditions.

In conclusion, cancerous CD168 expression can be used as a prognostic marker for gastric cancer owing to its interaction with HA. Combination analysis with immunological or genetic markers including CD168 appears to be appropriate to predict patient outcome.

## Competing interests

The authors declare that they have no competing interests.

## Authors' contributions

SI carried out the immuno histopathological studies and performed the statistical analysis. SN participated in its design and coordination. All authors read and approved the final manuscript.

## Pre-publication history

The pre-publication history for this paper can be accessed here:

http://www.biomedcentral.com/1471-2407/11/106/prepub

## Supplementary Material

Additional file 1 Table S1: Patients' information Click here for file

Additional file 2 Table S2: Correlation between CD168 positivity and clinical factors Click here for file

Additional file 3 Table S3: Univariate and multivariate analysis of survival with clinical factors including CD168 expression Click here for file
