# Supplementary material for: Prognostic impact of CD168 expression in gastric cancer
Source: BMC Cancer. 2011 Mar 24;11:106. doi: 10.1186/1471-2407-11-106 (PMC3076262; doi:10.1186/1471-2407-11-106)
Supplement: Additional file 1 — Table S1: Patients' information [file 1471-2407-11-106-S1.DOCX]

　Table１． Patients’ information

Gender 　 Male 　 　　　 135

　 　　　　Female 　　　　61

Age 　　　　　　　　63 (43-87)

Operation Total gastrectomy 66

　 　　　　Distal gastrectomy 107

　 　　　　Proximal gastrectomy 23

Stage 　　　　　　　　　I 89

　 　　　　　　　　　II 27

　 　　　　　　　　　III 43

　 　　　　　　　　　IV 37

Histology Differentiated 106

　 　　　　Undifferentiated 90

CD168 positivity yes 　　　　57

　 　 　　　　no 　 　　　　139
