# Supplementary material for: Prognostic impact of CD168 expression in gastric cancer
Source: BMC Cancer. 2011 Mar 24;11:106. doi: 10.1186/1471-2407-11-106 (PMC3076262; doi:10.1186/1471-2407-11-106)
Supplement: Additional file 2 — Table S2: Correlation between CD168 positivity and clinical factors [file 1471-2407-11-106-S2.DOCX]

Table 2. Correlation between CD168 positivity and clinical factors

Clinical factors 　 　 CD168 positivity 　　　　 p value

　 　 　 　　　　　　　yes n=57 　　　no n=139

Gender 　　　　Male 　　　　39 　　　　　96 N.S.

　 　　　　Female 　　　　18 　　　　　43

Tumor depth T1 12 　　　　　69

　 　　　　T2 21 　　　　　42 p<0.01

　 　　　　T3- 23 　　　　　28

Nodal involvement yes 33 　　　　　55 p<0.05

　 　　　　　　　　no 24 　　　　　84

Lymphatic invasion yes 42 　　　　　75 p<0.01

　 　　　　　　　　no 15 　　　　　64

Venous invasion 　　　　yes 33 　　　　　44 p<0.01

　 　　　　　　　　no 23 　　　　　95

Histology Differentiated 24 　　　　　67 N.S.

　 　　　　Undifferentiated 　 33 　　　　　72
