# Supplementary material for: Prognostic impact of CD168 expression in gastric cancer
Source: BMC Cancer. 2011 Mar 24;11:106. doi: 10.1186/1471-2407-11-106 (PMC3076262; doi:10.1186/1471-2407-11-106)
Supplement: Additional file 3 — Table S3: Univariate and multivariate analysis of survival with clinical factors including CD168 expression [file 1471-2407-11-106-S3.DOCX]

Table 3 Univariate and multivariate analysis of survival with clinical factors including

CD168 expression

Factors 　 　　　　　Univariate 　 　 Multivariate

　 　 　　　　　　p value 　　　p value Hazard ratio 95% CI

Age 　　　　　　<0.01 <0.01 　0.33 　　　0.18-0.62

Gender 　　　　　　<0.05 n.s. 　0.91 　　　0.7-2.3

CD168 　　　　　　<0.01 <0.05 　0.48 　　　0.27-0.85

Tumor depth 　　　　　　<0.01 <0.01 　0.42 　　　0.23-0.79

Nodal involvement 　　<0.01 <0.01 　0.24 　　　0.11-0.54

Tumor length 　　　　　　<0.05 　 <0.05 　0.3 　　　0.24-0.93
